# Rare Progressive Edema From a Secondary Capillary Leak Syndrome in Autoimmune Disease

**DOI:** 10.7759/cureus.109611

**Published:** 2026-05-25

**Authors:** Lucy Shang, John Weng, Sarah Lane, May Zaw

**Affiliations:** 1 Internal Medicine, Icahn School of Medicine at Mount Sinai, New York City, USA; 2 Anesthesiology, Icahn School of Medicine at Mount Sinai, New York City, USA

**Keywords:** anasarca, autoimmune, capillary leak syndrome, noncardiogenic pulmonary edema, progressive edema

## Abstract

Secondary capillary leak syndrome (SCLS) is a rare and frequently underrecognized cause of progressive edema, hypoalbuminemia, and noncardiogenic pulmonary edema. We present a case of a 55-year-old man with seronegative rheumatoid arthritis and antisynthetase syndrome on chronic immunosuppression who developed progressive bilateral lower-extremity edema and pulmonary edema in the absence of cardiac, renal, or hepatic disease. Extensive evaluation excluded alternative etiologies. The patient met diagnostic criteria for SCLS and demonstrated clinical improvement with albumin-assisted diuresis. This case highlights an underrecognized autoimmune-mediated phenotype of SCLS and underscores the importance of recognizing progressive edema without shock as a manifestation of SCLS.

## Introduction

Capillary leak syndrome (CLS) is a rare disorder characterized by pathologic endothelial hyperpermeability resulting in extravasation of protein-rich plasma into the interstitial space [1]. Loss of intravascular fluid and albumin leads to edema, hypoalbuminemia, and reduced effective circulating volume despite total body fluid overload. In severe cases, endothelial dysfunction may result in hypotension, shock, hemoconcentration, compartment syndromes, and multiorgan failure [1,2]. CLS is traditionally divided into idiopathic systemic CLS (Clarkson disease) and secondary CLS (SCLS), which occurs in association with autoimmune disease, infection, malignancy, transplantation, or medications [2].

The underlying pathophysiology of CLS remains incompletely understood but is believed to involve immune-mediated endothelial injury and dysregulation of vascular permeability pathways. Experimental and clinical studies have implicated inflammatory cytokines, complement activation, vascular endothelial growth factor (VEGF), angiopoietin-2, and leukocyte-endothelial interactions in the disruption of endothelial barrier integrity [1,3]. A central feature appears to be dysfunction of endothelial adherens junctions, particularly involving vascular endothelial (VE)-cadherin, a key protein responsible for maintaining intercellular junction stability and vascular permeability [4]. Cytokine-mediated internalization and redistribution of VE-cadherin increases endothelial permeability and promotes plasma extravasation into the interstitial compartment [4].

Classic idiopathic systemic CLS typically presents with abrupt episodes of hypotension, hemoconcentration, and hypoalbuminemia [1]. In contrast, secondary CLS often follows a more indolent course and may present predominantly with progressive edema and pulmonary congestion without overt shock or hemoconcentration [2]. This more subtle phenotype frequently leads to diagnostic delay because the presentation overlaps substantially with more common causes of edema, including heart failure, nephrotic syndrome, and cirrhosis.

Autoimmune diseases and immune-modulating therapies have emerged as important contributors to secondary CLS [3]. Case series have demonstrated associations between CLS and connective tissue diseases, particularly Sjögren syndrome and inflammatory myopathies, with a notable prevalence of anti-SSA/Ro antibodies [3]. These autoantibodies have been implicated in endothelial injury and vasculitic processes, raising the possibility of direct antibody-mediated vascular dysfunction. Additionally, immunotherapies such as interleukin-2 and monoclonal antibodies, including rituximab, have been associated with cytokine-mediated endothelial injury and vascular leak [4,5]. Tacrolimus-associated endothelial toxicity and complement activation have also been described in isolated reports of secondary CLS [6].

We present a case of secondary CLS in a patient with antisynthetase syndrome and seronegative rheumatoid arthritis receiving chronic immunosuppressive therapy who developed progressive edema and noncardiogenic pulmonary edema in the absence of cardiac, renal, or hepatic disease. This case highlights the diagnostic challenges posed by the indolent autoimmune-associated phenotype of secondary CLS and emphasizes the importance of recognizing endothelial hyperpermeability syndromes in patients with otherwise unexplained edema.

## Case presentation

A 55-year-old man with a history of seronegative rheumatoid arthritis and antisynthetase syndrome complicated by interstitial lung disease (stable since 2020) presented with progressive bilateral lower-extremity edema and dyspnea. His medical history was notable for chronic immunosuppression with prednisone, mycophenolate mofetil, tacrolimus, and rituximab. Additional comorbidities included gastroesophageal reflux disease, obesity, osteopenia, and benign prostatic hyperplasia status post transurethral resection of the prostate.

Four to six weeks prior to admission, the patient developed gradual, symmetric lower-extremity swelling that progressed to the point that he could no longer fit into his shoes. This was accompanied by worsening fatigue and joint pain. Several days before presentation, he developed a cough and exertional dyspnea.

**Figure 1:**
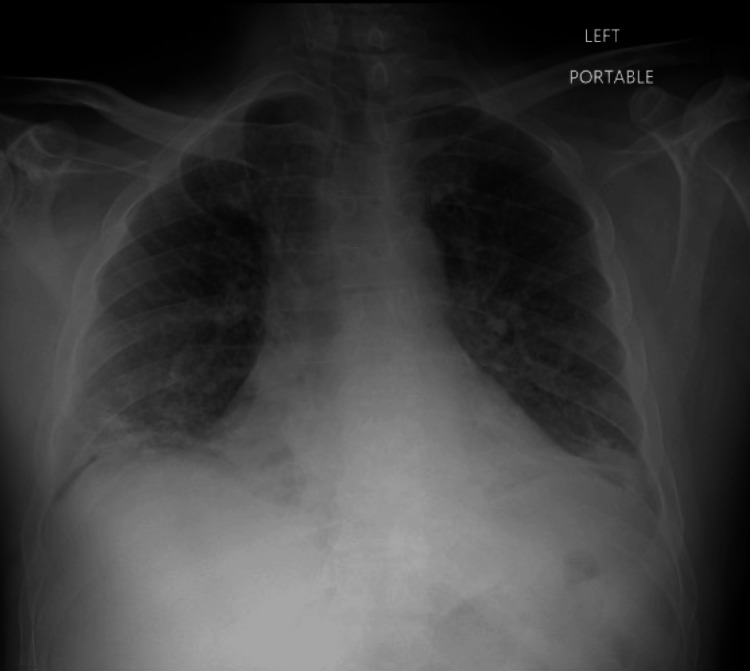
X-ray chest showing interstitial pulmonary edema

Chest radiography demonstrated interstitial pulmonary edema (Figure 1). Transthoracic echocardiography revealed normal biventricular size and function with a left ventricular ejection fraction of 63%, no valvular abnormalities, and no diastolic dysfunction. Bilateral lower-extremity venous Doppler studies were negative for thrombosis.

Laboratory evaluation was notable for decreased albumin, hemoglobin, and hematocrit levels, along with markedly increased inflammatory markers (Table 1). On chart review, the patient had previous positive autoimmune serologies, including anti-SSA/Ro antibodies and anti-Jo-1 antibodies. Liver enzymes were normal, with no imaging or laboratory evidence of cirrhosis. Urine protein-to-creatinine ratio was normal, effectively excluding nephrotic syndrome. The patient’s blood pressure was within the low-normal range throughout admission.

**Table 1:** Laboratory evaluation on presentation *Reference ranges may vary based on sex and institutional standards and provided here as standard adult ranges.

Laboratory Parameter	Patient Value	Reference Range	Interpretation
Albumin	2.3 g/dL	3.5–5.0 g/dL	Decreased
C-reactive protein (CRP)	125.7 mg/L	<10 mg/L	Increased
Hemoglobin	11.2 g/dL	12.0–16.0 g/dL*	Decreased
Hematocrit	34.70%	36–46%*	Decreased
Urine protein-to-creatinine ratio	0.2 g/day	<0.3 g/day	Normal

Given the recognized association between SCLS and hematologic malignancy, particularly plasma cell dyscrasias and lymphoproliferative disorders, evaluation for neoplastic etiologies was undertaken. Serum protein electrophoresis, urine protein electrophoresis, and serum immunofixation demonstrated no monoclonal gammopathy. Serum free light chains were not suggestive of a plasma cell disorder. Computed tomography imaging obtained during the admission showed no lymphadenopathy, splenomegaly, or evidence of occult solid-organ malignancy. Additionally, the patient denied constitutional symptoms, including fevers, night sweats, or unintentional weight loss, and laboratory evaluation revealed no unexplained cytopenias or peripheral blood abnormalities suggestive of an underlying hematologic process. Collectively, these findings made a neoplastic etiology for the patient’s presentation unlikely.

Given the combination of progressive anasarca, hypoalbuminemia without proteinuria, noncardiogenic pulmonary edema, and exclusion of alternative etiologies, the patient met criteria for SCLS, likely driven by immune-mediated endothelial dysfunction in the setting of autoimmune disease and rituximab treatment.

The patient was treated with intravenous albumin followed by high-dose intravenous furosemide (80 mg twice daily), resulting in improved diuresis, reduction in lower-extremity edema, and symptomatic improvement. He remained hemodynamically stable throughout hospitalization without progression to shock or acute kidney injury.

Given concern for ongoing immune-mediated endothelial dysfunction, the patient’s chronic immunosuppressive regimen was reassessed in multidisciplinary consultation with rheumatology. Tacrolimus levels remained within the therapeutic range and were not felt to warrant discontinuation. Rituximab was deferred during the acute hospitalization, while maintenance prednisone and mycophenolate mofetil were continued to avoid precipitating a flare of the underlying autoimmune disease. At outpatient follow-up, the patient demonstrated sustained improvement in edema and respiratory symptoms with stable renal and cardiopulmonary function and no recurrence of severe volume overload.

## Discussion

This case illustrates an often overlooked presentation of SCLS: slowly progressive edema and pulmonary congestion without hemoconcentration. Unlike idiopathic SCLS, secondary forms frequently lack dramatic hemodynamic collapse and instead manifest as persistent, unexplained volume overload with hypoalbuminemia that is poorly responsive to standard diuretic therapy.

Diagnostic considerations

The diagnosis of SCLS is one of exclusion and requires careful evaluation to rule out heart failure, nephrotic syndrome, cirrhosis, venous obstruction, protein-losing enteropathy, and neoplastic etiologies. Our patient met key diagnostic criteria: (1) progressive edema and pulmonary edema, (2) hypoalbuminemia without albuminuria, (3) preserved cardiac function, and (4) absence of alternative causes. Importantly, nephrotic syndrome was excluded by a normal urine protein-to-creatinine ratio, while echocardiography demonstrated normal systolic and diastolic function without evidence of elevated filling pressures. Similarly, hepatic dysfunction and cirrhosis were not supported by laboratory or imaging findings.

Given the established association between CLS and plasma cell dyscrasias or lymphoproliferative disorders, neoplastic etiologies were also considered. However, evaluation revealed no monoclonal gammopathy, constitutional symptoms, lymphadenopathy, splenomegaly, or imaging findings suggestive of occult malignancy. The absence of hemoconcentration in this patient is consistent with the more indolent and subacute tempo increasingly recognized in SCLS [2]. This distinction is clinically important because progressive edema without shock may delay recognition and lead clinicians to attribute symptoms solely to more common cardiopulmonary or renal processes.

Autoimmune and immune-mediated pathophysiology

Growing evidence supports immune-mediated endothelial injury as a central mechanism in secondary CLS [1]. Endothelial barrier dysfunction appears to result from inflammatory cytokines, complement activation, and disruption of intercellular junction proteins responsible for maintaining vascular integrity. Several studies have implicated vascular endothelial growth factor (VEGF), angiopoietin-2, tumor necrosis factor-α, and other inflammatory mediators in promoting endothelial hyperpermeability and plasma extravasation [1,3].

Case series have demonstrated associations between CLS and autoimmune diseases, particularly Sjögren syndrome and inflammatory myopathies, with a notable prevalence of anti-SSA/Ro antibodies [3]. These antibodies have been implicated in endothelial injury and vasculitic processes, raising the possibility of direct antibody-mediated endothelial dysfunction. Our patient’s history of antisynthetase syndrome, inflammatory arthritis, and positive anti-SSA/Ro antibodies supports the hypothesis that chronic autoimmune activation contributed to sustained endothelial permeability.

Cytokine-driven permeability changes also play a critical role in the pathogenesis of vascular leak. Experimental models have demonstrated that inflammatory cytokines and immune therapies disrupt endothelial adherens junctions, particularly through internalization of VE-cadherin, a key transmembrane protein responsible for maintaining endothelial junctional stability [4]. Interleukin-2-induced vascular leak has been shown to occur via VE-cadherin redistribution and endothelial barrier dysfunction, providing a mechanistic framework that may extend to other immune-activating therapies [4]. Collectively, these findings support the concept that chronic immune dysregulation may predispose susceptible patients to a more indolent but persistent capillary leak phenotype.

Rituximab and tacrolimus as potential contributors

Rituximab, a monoclonal anti-CD20 antibody widely used in autoimmune disease and hematologic malignancy, has been reported to be associated with CLS through cytokine release and complement activation [5]. Although previously reported cases typically involve acute presentations with hypotension and shock physiology, drug-induced CLS may preferentially involve pulmonary manifestations and noncardiogenic pulmonary edema. In our patient, chronic rituximab exposure in the setting of active systemic autoimmune inflammation may have contributed to persistent endothelial dysfunction rather than an abrupt cytokine-release syndrome.

Tacrolimus has also been implicated in isolated reports of CLS, potentially through endothelial toxicity and complement-mediated vascular injury [6]. However, most reported cases have occurred in the setting of supratherapeutic tacrolimus levels or transplant-associated thrombotic microangiopathy. In our patient, tacrolimus levels remained within the therapeutic range, and there was no evidence of hemolysis, thrombocytopenia, or renal dysfunction suggestive of thrombotic microangiopathy, making tacrolimus a less likely primary driver. Nevertheless, chronic calcineurin inhibitor exposure may have contributed to baseline endothelial vulnerability in combination with the patient’s underlying autoimmune disease and biologic therapy.

Management implications

Management of SCLS is primarily supportive and differs substantially from the treatment of cardiogenic edema or isolated volume overload states. A central pathophysiologic feature of CLS is the coexistence of total body fluid excess with relative intravascular depletion due to transcapillary albumin and plasma leakage. As a result, aggressive diuresis alone may worsen effective circulating volume depletion, impair renal perfusion, and exacerbate hypotension.

In our patient, standard diuretic escalation initially produced limited improvement, raising concern for impaired intravascular refill despite extensive edema. Albumin-assisted diuresis was therefore employed to restore oncotic pressure and facilitate mobilization of interstitial fluid into the vascular compartment before loop diuretic administration. This strategy resulted in improved diuresis, reduction in edema, and symptomatic recovery without hemodynamic compromise or acute kidney injury. The favorable response further supports the underlying physiology of endothelial hyperpermeability and intravascular oncotic depletion rather than primary cardiac or renal failure.

Intravenous immunoglobulin (IVIG) has demonstrated benefit in idiopathic systemic CLS and in some refractory secondary cases, likely through immunomodulatory effects including cytokine suppression and stabilization of endothelial function [7]. However, its role in isolated, nonrecurrent secondary CLS remains uncertain. In our patient, conservative supportive management and reassessment of immunomodulatory therapy were sufficient to achieve clinical improvement without escalation to IVIG or additional immunosuppressive interventions.

## Conclusions

This case highlights secondary capillary leak syndrome as a rare but clinically significant cause of progressive edema and noncardiogenic pulmonary edema in patients with autoimmune disease receiving chronic immunomodulatory therapy. Unlike classic idiopathic systemic capillary leak syndrome, secondary forms may follow a more indolent clinical course characterized by gradual anasarca, hypoalbuminemia, and low-normal blood pressure without profound hypotension or hemoconcentration. As demonstrated in this case, these atypical presentations can closely mimic more common cardiopulmonary, renal, or hepatic causes of edema and may therefore result in substantial diagnostic delay. Our patient presented with progressively worsening edema, hypoalbuminemia without proteinuria, elevated inflammatory markers, and pulmonary congestion despite preserved cardiac function and absence of significant renal, hepatic, or neoplastic disease. The case underscores the importance of maintaining a broad differential diagnosis when evaluating refractory edema that is disproportionate to objective evidence of heart failure or nephrotic syndrome. Careful exclusion of alternative etiologies, combined with recognition of endothelial hyperpermeability physiology, was essential in establishing the diagnosis. This report also contributes to the growing body of literature supporting an immune-mediated pathophysiology of secondary CLS. Prior studies have demonstrated associations between CLS and connective tissue diseases, inflammatory myopathies, anti-SSA/Ro antibodies, and immune-modulating therapies, including rituximab. In our patient, chronic autoimmune activation together with prolonged exposure to biologic and calcineurin inhibitor therapy likely contributed to sustained endothelial dysfunction and vascular leak. This case supports the hypothesis that chronic immune dysregulation may underlie a more subacute and persistent phenotype of secondary CLS distinct from the abrupt shock-associated presentation of idiopathic Clarkson disease. From a therapeutic perspective, this case highlights the physiologic distinction between total body fluid overload and effective intravascular volume depletion in endothelial hyperpermeability syndromes. Conventional escalation of diuretic therapy alone may therefore be ineffective and can worsen intravascular depletion and organ hypoperfusion. In contrast, albumin-assisted diuresis in our patient resulted in improved mobilization of interstitial fluid and symptomatic recovery without hemodynamic compromise or renal injury, emphasizing the importance of individualized volume management strategies guided by underlying pathophysiology.

As the use of biologic and immune-modulating therapies continues to expand across rheumatologic and autoimmune diseases, clinicians should maintain increased awareness of secondary CLS as a potential complication of chronic immune dysregulation and endothelial injury. Earlier recognition of atypical presentations may reduce unnecessary diagnostic evaluation, facilitate more appropriate supportive management, and ultimately reduce preventable morbidity in medically complex patients.
